# Defining the Role for Topically Administered Tranexamic Acid in Panniculectomy Surgery

**DOI:** 10.1093/asjof/ojac033

**Published:** 2022-05-05

**Authors:** Jason M Weissler, Doga Kuruoglu, Cristina Salinas, Nho V Tran, Minh-Doan T Nguyen, Jorys Martinez-Jorge, Uldis Bite, Christin A Harless, Aparna Vijayasekaran, Basel Sharaf

## Abstract

**Background:**

Abdominal panniculectomy after weight loss is a commonly performed procedure with high patient satisfaction yet continues to have a high post-operative complication profile. Several risk-reducing surgical approaches, such as preservation of Scarpa’s fascia, use of tissue adhesives, and progressive tension suture techniques have been described. However, the use of tranexamic acid (TXA) has not been previously reported in panniculectomy surgery.

**Objectives:**

To improve the safety and predictability of this procedure, the authors investigate whether the use of topically administered TXA during panniculectomy surgery reduces seroma, hematoma, and drain duration.

**Methods:**

Consecutive patients who underwent panniculectomy (January 2010 to January 2022) were retrospectively reviewed. Outcome measures included hematoma requiring surgical evacuation, seroma requiring percutaneous aspiration, and drain duration. Patients with thromboembolic diseases and those taking anticoagulation/antiplatelet medications were excluded. Patients who had received TXA were compared with a historical control group who had not received TXA.

**Results:**

A total of 288 consecutive patients were included. Topical TXA was administered in 56 (19.4%) cases. The mean (standard deviation [SD]) follow-up was 43.9 (37.4) months (3.7 years). The median (range) resection weight was 2.6 kg (0.15-19.96 kg). Regarding seroma and hematoma formation, the use of TXA did not reduce the likelihood of developing seroma or hematoma (odds ratio [OR] = 1.7, 95% CI [0.56- 4.8], *P* = 0.38 and OR = 2.1, 95% CI [0.4-11.8], *P* = 0.42), respectively. The mean (SD) duration of drains was slightly lower in the TXA group (18.1 [12.1] days vs 19.8 [13.9] days); however, this difference was not statistically significant, albeit clinically significant.

**Conclusions:**

As the use of TXA in plastic surgical procedures continues to expand, the utility of TXA in panniculectomy and abdominoplasty has not been elucidated. Although previous studies report hematoma and seroma risk reduction, the use of TXA was not associated with a statistically significant reduction in seroma, hematoma, or drain duration following panniculectomy surgery. Prospective, randomized controlled studies on the use of TXA in body contouring are needed.

**Level of Evidence: 3:**

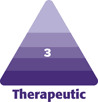

As the incidence of obesity continues to increase globally, the demand for bariatric surgery and post-bariatric body contouring surgery has become more prevalent. Following weight loss surgery, patients experience significant improvement in health-related conditions, including diabetes, hypertension, and sleep apnea. However, excess skin and subcutaneous tissue laxity affecting different parts of the body including the abdomen ensues. Resultant intertriginous rashes and infections, difficulty with maintaining personal hygiene, functional impairment, and psychological distress may affect patients negatively.^1-3^

As such, up to one-third of weight loss patients ultimately pursue post-weight loss body contouring such as panniculectomy and abdominoplasty.^4^ Benefits of undergoing panniculectomy have been well documented in the literature and include improved quality of life, improved hygiene, decreased intertriginous infection rates, and improved psychosocial well-being.^1,2^ Despite significantly improved patient satisfaction following panniculectomy, this procedure has historically been associated with a higher complication profile, including postoperative wound infection, dehiscence, seroma, hematoma, and venous thromboembolism (VTE). Furthermore, Lesko et al demonstrated that patients who have undergone panniculectomy were nearly 3-times more likely than abdominoplasty patients to develop postoperative complications after adjusting for BMI, American Society of Anesthesiologists (ASA) class, mesh use, and concurrent surgery.^5^

With overall complication rates ranging from 21% to 56%, previous authors have analyzed the specific complications associated with panniculectomy and have published on the importance of modifiable risk factors to mitigate any increased risk, such as BMI reduction and smoking cessation.^4,6-8^ Given that panniculectomy is an elective surgery, reducing adverse events such as hematoma, seroma, and infection is imperative. While the risk of bleeding and seroma exists for all surgical procedures, reoperations for hematoma evacuations, repeated in-office seroma aspirations, and prolonged drain duration have been demonstrated to negatively impact patient experiences.^9-14^ To decrease the incidence of postoperative seromas, affecting as many as 30% of patients undergoing panniculectomy, some surgeons depend upon drains to mitigate seroma formation.

Additionally, the introduction of various surgical techniques, such as progressive tension sutures and pharmacologic adjuncts, such as fibrin sealants, has been used to further combat postoperative seromas and bleeding risk. Despite these previously demonstrated adjuncts, the use of tranexamic acid (TXA) in panniculectomy surgery has not been previously studied. TXA functions to block the conversion of plasminogen to plasmin and prevents the degradation of fibrin clots by plasmin. TXA also blocks plasmin-induced platelet activation, which preserves platelets for the formation of clot. Furthermore, TXA has also been shown to have anti-inflammatory properties by inhibiting plasmin activation.^15,16^ This antifibrinolytic medication has gained increasing recognition in plastic surgery as a safe and clinically effective medication capable of minimizing blood loss, reducing seroma formation, bruising, and postoperative edema in procedures such as craniofacial surgery, facial aesthetics, breast reconstruction, and aesthetic body contouring procedures.^15-21^ Although its safety and efficacy have been well documented in the surgical literature, there have been no published data on its use in panniculectomy surgery.

As the role of TXA within plastic surgery has become better defined, further contribution to the expanding body of literature is imperative. To improve the safety and predictability of panniculectomy surgery, the authors aim to study the effectiveness and safety profile of topically administered TXA among panniculectomy patients to ascertain whether it reduces seroma and hematoma formation while allowing for shorter drain duration.

## METHODS

### Study Design and Patient Selection

With the Mayo Clinic IRB approval, a retrospective chart review of consecutive patients over 18 years of age who underwent abdominal panniculectomy at our institution between January 1, 2010, and January 1, 2022, was performed. Patients who had previously undergone panniculectomy, abdominoplasty, or circumferential belt lipectomy and patients with less than 1-month follow-up were excluded. Furthermore, patients with coagulopathies, history of thromboembolism, or bleeding diatheses were excluded from the review. BMI at the time of panniculectomy was classified according to the World Health Organization (WHO) Obesity Classification. Patients with BMI < 30 kg/m^2^ were considered nonobese.

Procedures performed only by plastic surgeons at our institution were included. Common Procedural Terminology (CPT) codes (15830 without an adjunct CPT code 15847 [abdominoplasty]) were used and confirmed by operative note review. Demographics including gender, age, and BMI at the time of panniculectomy and comorbidities including smoking status, history of VTE, hypertension, diabetes mellitus, dyslipidemia, coronary artery disease; history of bariatric surgery; and history of laparotomy were documented. Surgical details were recorded and included type of panniculectomy (transverse skin excision vs inverted-T [fleur-de-lis]), concomitant ventral hernia repair or concomitant suction-assisted lipectomy. Additionally, the following intraoperative details were collected: the intraoperative use of topical TXA, drain use, and resection weight. Drain duration was captured and defined as the difference between the date when the last remaining drain was removed and the date of surgery (reported in days). Patients who received TXA were compared with a historical control group who had not received TXA within the same cohort.

The primary outcomes were (1) hematoma necessitating surgery and (2) clinically significant seroma, defined as the development of symptoms (palpable fluid collection and increased pain) mandating percutaneous aspiration or drainage. Secondary outcome measures included (1) surgical site infection (SSI) defined according to the Centers for Disease Control definition and (2) wound healing complications requiring surgery. Minor complications were wounds or incisional issues which were managed with dressings.

### Surgical Technique

All surgeries were performed by board-certified plastic surgeons at our institution. The decision to proceed with a fleur-de-lis skin excision was determined preoperatively if there was excess upper/vertical soft tissue redundancy. The lower abdominal incision was made in a typical fashion with dissection to the rectus abdominis fascia and limited to the abdominal skin redundancy. Lateral abdominal wall perforators were preserved. If necessary, discontinuous undermining was performed when a fleur-de-lis excision was planned. A multi-layered closure was performed using a combination of absorbable monofilament sutures to reapproximate Scarpa’s fascia, deep dermis, and skin. Progressive tension sutures were not used for any patients in this study. At least two 15-French round channel drains were placed in the undermined region and were maintained on bulb suction until the drains met the criteria, defined as output less than 30 cc each day for 2 consecutive days. 

The use of TXA was introduced into our panniculectomy procedures in April 2018 after noticing promising results in our breast reconstruction practice. The decision to use TXA was surgeon dependent and was not initially used in consecutive patients as its use was slowly incorporated into our practice over the past 5 years. When used, 3 g of TXA in 75 mL of NaCl 0.9% was delivered topically, both directly to the wound surfaces (moistening the tissue) and infiltrated retrograde through the placed drains following the completion of wound closure. Drains were placed to bulb suction 1 hour after closure to prevent inadvertent removal of the instilled TXA. An abdominal binder was placed in all patients, and patients were instructed to apply it during ambulation. In select patients, based on preoperative Caprini risk assessment score, deep venous thrombosis prophylaxis was prescribed. One dose of weight-appropriate intravenous first-generation cephalosporin was administered within 1 hour before incision. The use of postoperative prophylactic antibiotics was surgeon dependent but, if used, was discontinued at the time of drain removal. 

### Statistical Analysis

Descriptive statistics were used to present the data. Univariate logistic regression analyses were performed to compare patient characteristics as well as the primary and secondary outcome measures in both groups. Univariate and multivariable (when applicable) logistic regression analyses were performed to determine associations between variables and postoperative complications. The effect of topical TXA on the duration of drains was assessed. Analyses were performed with JMP Statistical Software (v16) (JMP, SAS Institute Inc., Cary, NC). An α error of 0.05 was used, and values of *P* < 0.05 were considered statistically significant. Sample size calculations were also performed, which determined that at least 262 panniculectomies needed to be included to achieve a power of 0.80, assuming a 4:1 ratio and a 2-sided significance level of 5% between the historical control (non-TXA) vs TXA group.

## RESULTS

Two hundred eighty-eight consecutive patients were reviewed. The mean (standard deviation [SD]) age and BMI were 51.2 (12.8) years and 32.9 (7.1) kg/m^2^, respectively. Topical TXA was used intraoperatively in 56 (19.4%) patients, whereas the non-TXA group consisted of 232 (81.6%) patients. The mean (SD) follow-up was 43.9 (37.4) months (3.7 years). The median (range) resection weight was 2.6 kg (0.15-19.96 kg). Table 1 summarizes the patient and clinical characteristics of our cohort as well as the TXA and non-TXA treatment groups.

**Table 1.** Patient Demographics and Clinical Characteristics

Demographics and characteristics	Overall cohort (n)	No TXA group (n)	TXA group (n)	*P*-value*
Patients	288	232	56	n/a
Mean age at panniculectomy (SD), y	51.2 (12.8)	51.7 (12.6)	49.2 (13.3)	0.19
Mean BMI at panniculectomy (SD), kg/m^2^	32.9 (7.1)	33.2 (7.4)	31.6 (5.7)	0.13
Obesity status at panniculectomy	n/a	n/a	n/a	0.34
Nonobese	106 (36.8)	82 (35.3)	24 (42.9)	n/a
Class I	85 (29.5)	67 (28.9)	18 (32.1)	n/a
Class II	56 (19.5)	46 (19.8)	10 (17.9)	n/a
Class III	39 (13.5)	35 (15.1)	4 (7.1)	n/a
Missing	2 (0.7)	2 (0.9)	0	n/a
Smoking status	n/a	n/a	n/a	0.31
Never smoker (%)	170 (59)	134 (57.8)	36 (64.3)	n/a
Former smoker (%)	103 (35.8)	84 (36.2)	19 (33.9)	n/a
Active smoker (%)	15 (5.2)	14 (6)	1 (1.8)	n/a
Hypertension (%)	124 (43.1)	98 (42.2)	26 (46.4)	0.57
Diabetes mellitus (%)	83 (28.8)	71 (30.6)	12 (21.4)	0.17
Dyslipidemia (%)	138 (47.9)	111 (47.9)	27 (48.2)	0.96
Coronary artery disease (%)	36 (12.5)	30 (12.9)	6 (10.7)	0.65
History of bariatric surgery (%)	184 (63.9)	150 (64.7)	34 (60.7)	0.67
History of open abdominal surgery (%)	207 (71.9)	166 (71.6)	41 (73.2)	0.8
Concurrent ventral hernia repair (%)	86 (29.9)	75 (32.3)	11 (19.6)	0.063
Concurrent SAL (%)	40 (13.9)	26 (11.2)	14 (25)	0.007^**^
Fleur-de-lis (%)	122 (42.4)	97 (41.8)	25 (44.6)	0.7
Median resection weight (range), kg	2.6 (0.15-19.96)	2.6 (0.15 - 14.6)	2.3 (0.37-19.96)	0.34
Median duration of drains in place (IQR), d	16 (12)	17 (12)	15 (11.5)	0.4
Mean follow-up (SD), mo	43.9 (37.4)	52.5 (36.7)	8.2 (6.1)	<0.0001^**^

*Comparisons were performed using logistic regression.

^**^Statistical significance. IQR, interquartile range; SAL, suction-assisted lipectomy; SD, standard deviation; TXA, tranexamic acid.

Overall, the rate of primary complications was 8.3% (seroma requiring drainage [n = 18, 6.2%]; hematoma requiring surgical evacuation [n = 6, 2.1%]). The rate of secondary complications was 14.6% (SSI [n = 32, 11.1%]; wound requiring surgical intervention [n = 10, 3.5%]). Wound-related problems that were managed by dressing care occurred in 43 (14.9%) patients. Complications are summarized in Table 2. The mean (SD) duration of drains was slightly lower in the TXA group [18.1 (12.1) days vs 19.8 (13.9) days]; however, this difference was not statistically significant, albeit clinically significant. TXA-related complications were not observed, including venous thromboembolic events or seizures.

**Table 2.** Major Complications

Complications	Overall n (%)	No TXA group n (%)	TXA group n (%)	*P*-value*
Primary complications				
Seroma^a^	18 (6.2)	13 (5.6)	5 (8.9)	0.38
Hematoma^a^	6 (2.1)	4 (1.7)	2 (3.6)	0.42
Secondary complications				
Surgical site infection	32 (11.1)	30 (12.9)	2 (3.6)	0.06
Wound^a^	10 (3.5)	9 (3.9)	1 (1.8)	0.45

^a^Requiring aspiration, drainage, evacuation, or surgical intervention.

*Comparisons were performed using logistic regression. TXA, tranexamic acid.

With regard to seroma formation, the univariate logistic regression analysis demonstrated that the use of TXA did not reduce the likelihood of developing a seroma (odds ratio [OR] = 1.7, 95% CI [0.56-4.8], *P* = 0.38). However, a history of hypertension (OR = 3.7, 95% CI [1.3-10.8], *P* = 0.02) and dyslipidemia (OR = 4.1, 95% CI [1.3-12.8], *P* = 0.015) were both significantly associated with a higher chance of developing a postoperative seroma. Our univariate logistic regression analysis also demonstrated that the use of TXA did not significantly decrease the odds of hematoma formation (OR = 2.1, 95% CI [0.4-11.8], *P* = 0.42). Furthermore, the univariate analysis demonstrated that patients undergoing fleur-de-lis panniculectomies were significantly more likely to develop a hematoma when compared with transverse-only incision patterns (4.9% in fleur-de-lis vs 0% in transverse incision, *P* = 0.0012). Tables 3 and 4 summarize the logistic regression analyses for the primary outcome measures.

**Table 3.** Univariate Logistic Regression Analysis to Identify Predictors of Seroma

Predictors of seroma	Unadjusted OR	95% CI	*P-*value
Age at time of panniculectomy	1.001	0.96-1.04	0.96
BMI at time of panniculectomy	1.02	0.96-1.09	0.52
Smoking status			
Never smoker	Ref	Ref	Ref
Former smoker	1.7	0.62-4.69	0.3
Active smoker	3.1	0.6-16.2	0.18
Hypertension	3.7	1.3-10.8	0.02*
Diabetes mellitus	0.95	0.33-2.75	0.92
Dyslipidemia	4.1	1.3-12.8	0.015*
Coronary artery disease	1.4	0.4-5.2	0.58
History of bariatric surgery	1.1	0.4-3.07	0.83
History of open abdominal surgery	0.5	0.18-1.22	0.12
Concurrent ventral hernia repair	1.54	0.58-4.1	0.39
Concurrent SAL	0.35	0.05-2.7	0.31
Fleur-de-lis	0.86	0.3-2.3	0.76
The use of tranexamic acid	1.7	0.56-4.8	0.38
Resection weight	1	0.9997-1.0002	0.62

*Statistically significant variable. OR, odds ratio; Ref, reference variable; SAL, suction-assisted lipectomy.

**Table 4.** Univariate Logistic Regression Analysis to Identify Predictors of Hematoma

Predictors of hematoma	Unadjusted OR	95% CI	*P-*value
Age at time of panniculectomy	1.03	0.97-1.1	0.35
BMI at time of panniculectomy	1.02	0.92-1.13	0.73
Smoking status			
Never smoker	Ref	Ref	Ref
Former smoker	1.7	0.33-8.4	0.53
Active smoker	0	NA	0.99
Hypertension	2.7	0.49-15	0.24
Diabetes mellitus	0.49	0.06-4.24	0.48
Dyslipidemia	2.2	0.4-12.3	0.35
Coronary artery disease	1.4	0.16-12.4	0.77
History of bariatric surgery	2.8	0.33-24.5	0.3
History of open abdominal surgery	0.78	0.14-4.3	0.78
Concurrent ventral hernia repair	1.18	0.2-6.6	0.85
Concurrent SAL	0	NA	0.18
Fleur-de-lis	Inf	NA	0.0012*
The use of tranexamic acid	2.1	0.4-11.8	0.42
Resection weight	1	0.9992-1.0007	0.94

*Statistically significant variable. α ratio per 1-year increase. Inf, infinity; NA, not available; OR, odds ratio; Ref, reference variable; SAL, suction-assisted lipectomy.

With regard to the secondary outcomes, on univariate analyses, TXA administration did not significantly impact the development of SSI (OR = 0.25, 95% CI [0.058-1.077], *P* = 0.06) or the risk of postoperative wounds requiring surgical intervention (OR = 0.45, 95% CI [0.06-3.6], *P* = 0.45) although the rates of SSI and wound were both lower in the TXA group. Moreover, active smokers were found to have a significantly higher likelihood of having SSI compared with former smokers (OR = 5.9, 95% CI [1.44-24.1], *P* = 0.014); increased BMI (OR = 1.06 per 1 kg/m^2^ increase, 95% CI [1.01-1.11], *P* = 0.017) was significantly associated with increased odds of SSI. Notably, a concomitant ventral hernia repair (OR = 3.7, 95% CI [1.02-13.5], *P* = 0.047) increased the odds of developing a wound requiring surgical intervention.

Furthermore, a multivariable logistic regression model (area under the receiver operating characteristics [ROC] curve = 0.71) consisting of variables including the use of TXA, BMI, and smoking status demonstrated that: (1) active smokers compared with former smokers (adjusted OR, [aOR] = 5.1, 95% CI [1.19-21.7], *P* = 0.029) and (2) increased BMI (aOR = 1.05 per 1 kg/m^2^ increase, 95% CI [1.003-1.1], *P* = 0.039) were significantly associated with higher odds of SSI, whereas the use of TXA (aOR = 0.28, 95% CI [0.06-1.25], *P* = 0.051) had no significant impact on SSI.

## DISCUSSION

To date, there is a recognizable gap in the literature on the use of TXA in body contouring, specifically panniculectomy surgery. As such, the authors expanded upon our previously published work on the role of TXA in other procedures, including reconstructive breast surgery, liposuction, and reduction mammaplasty.^22,23^ To broaden the scope of its use and to contribute to the growing body of evidence, in the first study to date on its use, the authors investigated the use of topically delivered TXA in patients undergoing panniculectomy to study its effect on seroma formation, hematomas, and drain duration.

This study demonstrated that the use of topical TXA did not reduce the risk of seroma formation nor did it statistically significantly reduce total drain days following panniculectomy surgery. However, with regard to drain duration, drains were removed at an average of 1.7 days sooner among patients who had received TXA, which was clinically significant. Furthermore, hypertension and dyslipidemia were significantly associated with a higher likelihood of developing postoperative seromas. Additionally, patients undergoing fleur-de-lis panniculectomies were significantly more likely to develop a hematoma when compared with transverse-only incision patterns. With regard to other modifiable risk factors, active smokers and increased BMI conferred an increased risk for the development of SSIs.

Since its original introduction 60 years ago for the treatment of hereditary bleeding disorders and gynecologic-related bleeding, TXA has been thoroughly investigated in various surgical subspecialities with well-established safety and efficacy in randomized controlled trials.^18,24,25^ In recent years, the unique and valuable pharmacologic attributes of TXA have cultivated changes to the practice of plastic surgeons who have increasingly incorporated its use in facial aesthetic surgery, breast, and body procedures to mitigate bleeding, postoperative ecchymosis/edema, and drain duration.^18,24-26^

The mounting evidence on the efficacy of TXA in plastic surgery underscores a unique opportunity for surgeons to potentially reduce bleeding and seroma formation, while decreasing bruising, edema, and drain duration for a range of procedures, including body contouring surgery. Panniculectomy following significant fluctuations in weight has been demonstrated to significantly improve quality of life through improved skin hygiene, decreased infectious issues, and improved psychosocial well-being.^1,2^ Given the relatively high overall complication rates following panniculectomy, which has been cited to range between 21.2% and 56%,^5,7,27,28^ the investigation of additional interventions to lower the complication profile of panniculectomy is necessary.

Seroma formation, for example, remains a common complication following panniculectomy, with a reported rate as high as 30% in the literature and 6.2% within our cohort. Although seromas often resolve with time so long as drains are functioning appropriately, patients may require multiple office visits, more diagnostic imaging and associated cost, and pain associated with percutaneous aspirations. To decrease the incidence of seromas following panniculectomy, surgeons may use closed suction drains, despite evidence to suggest that drain placement may be unnecessary so long as empty space is managed appropriately with other modalities. For example, progressive tension sutures (PTS), which were first introduced and popularized by Drs Harlan Pollock and Todd Pollock in 2000 to promote empty-space obliteration, help minimize tension at the suture line and mitigate seroma rates in procedures such as abdominoplasties, lower body lifts, torsoplasty (bra-line lifts), and brachioplasties.^29,30^ Agochukwu-Nwubah and Patronella. published the results of PTS for the aforementioned procedures and demonstrated that the use of PTS is effective for reducing postoperative seromas in similar “flap” surgeries obviating the need for drain placement.^30-32^ Beyond suture techniques used to obliterate dead space, another surgical technique that has been described in the abdominoplasty patient population is the preservation of Scarpa’s fascia as a means of reducing seroma.^33^ This technique has been extensively described in the abdominoplasty literature; however, there is limited research on this technique in the panniculectomy patient population.

In addition to the previously described surgical techniques to minimize seroma formation, pharmacologic adjuncts, such as surgical adhesives or fibrin sealants, have been previously studied. Based on the principles of PTS and its ability to reduce shearing forces, a similar effect is described for methods in which the tissue layers are coated with surgical adhesives or fibrin sealants, which may be a safe and useful alternative or adjunct to PTS to minimize seromas following abdominoplasties.^34-38^

Furthermore, in an unpublished study by our group, the authors investigated the use of topically delivered TXA among 300 females undergoing implant-based breast reconstruction. In this study, patients who were administered topical TXA onto the mastectomy flaps and into the breast pocket were significantly less likely to develop postoperative seromas and had shorter drain duration when compared with patients who had not received TXA. Based on these findings, we recently investigated the use of topical, intravenous, and topical plus intravenous TXA among females undergoing reduction mammaplasty for symptomatic macromastia.^22^ Although the authors expected a similar reduction in seroma risk, the use of TXA, regardless of the delivery technique, did not impact nor decrease seroma risk following the reduction mammaplasty. 

In the current study, the authors found that topically delivered TXA did not have any statistically significant impact on the reduction of seroma, hematoma, or time to drain removal. There were no adverse events as a result of TXA administration, including VTE and seizures. Upon reflecting on the findings of this study, the authors expected to discover some risk reduction based on previously published literature from our institution as well as our anecdotal observation that patients were experiencing less seromas with earlier drain removal.^23^ Although we were unable to demonstrate the efficacy of TXA in reducing these complications, our findings coincide with previous publications which report no benefit from using pharmacologic adjuncts, such as fibrin sealants and tissue adhesives to reduce seroma formation or reduce drain days. Furthermore, although statistical significance was lacking with regard to drain duration, patients in the TXA cohort had drains removed almost 2 days sooner than patients who did not receive TXA. This certainly has merit in that perhaps we can minimize prolonged patient discomfort during recovery and minimize susceptibility to infectious complications as it pertains to drain use.

To date, there is no standardized way to deliver TXA among plastic surgery procedures. Thus, patients in our cohort who received TXA did not receive the 3 g in 75 cc of NaCl in a standardized fashion, as TXA was either sprayed directly onto the tissues throughout the entirety of the procedure or administered retrogradely through the drains near the conclusion of abdominal closure. Moving forward, it will be valuable in future work to ensure the delivery modality remains constant in order to more scientifically determine whether the delivery mode of TXA has an impact on its efficacy.

Notably, in the only published study on the pharmacokinetics of TXA in body contouring surgery, Ausen et al demonstrated that spraying the wound surface before closure creates a “homogenous film” of TXA on the entire wound surface when compared with retrograde/drain administration.^39^ These authors demonstrated more predictable pharmacokinetic metrics in the topical moistening group and concluded that spraying the wound surface should be considered in contrast to administering a bolus of topical TXA into the wound cavity (injecting retrograde through the drains) because the bolus of TXA is unlikely to homogenously coat the undermined tissue, causing unpredictable and variable pharmacokinetics. As such, in future work, we agree with Ausen et al in that we would advocate for topically moistening the wound surface rather than instillation of TXA as a topical bolus through the drains for a more predictable impact and potentially improved efficacy.^39^

While the ideal delivery modality and dose continue to be investigated, the expansive body of literature on the use of TXA has demonstrated safety and efficacy across a wide range of dosages and delivery modalities within many surgical subspecialities.^40^ Despite its favorable clinical properties, widespread use has yet to occur, perhaps in part due to unfamiliarity with its use, misconceptions regarding perceived untoward side effects, such as inducing thromboembolic events or seizures, and lack of prospective randomized data supporting its use in plastic surgery. Nonetheless, as evidenced in several meta-analyses, the local administration of TXA may provide sufficient drug concentrations at the wound surface with negligible risk of systemic adverse effects or increases in adverse events.^41-43^ Moreover, the lack of incorporation into practice may also be due to the perception that its use is cost prohibitive despite its reasonable cost profile. While the authors did not perform a formal cost analysis, the total cost at our institution for 3 g of TXA is roughly $25 and 1 g of intravenous TXA is approximately $14.

This is the first study to investigate the use of TXA in panniculectomy surgery. However, there are inherent limitations. The retrospective single-institution design yields the potential for selection bias. Also, although a sample size calculation was performed to achieve a power of 0.80, the low number of incidences of seroma (n = 18) and hematoma (n = 6) did not allow the authors to perform multivariable analyses to determine confounders or effect modifiers. Additionally, with all TXA-related research moving forward, it will be imperative to standardize the way in which TXA is administered with regard to delivery modality and dose. For topically administered TXA specifically, the way in which the TXA is delivered should be standardized to account for the heterogeneity of administration (directly spraying onto wound surfaces vs retrograde injection through the drain as a bolus). Establishing standardization within our practice will allow us to study the true clinical impact of TXA more scientifically in aesthetic or body contouring procedures. To gain further understanding of its role in body contouring procedures and to determine the optimal administrative modality and dose, we encourage all investigators to collaborate through large prospective, randomized, placebo-controlled trials.

## CONCLUSIONS

While plastic surgeons have become more familiar with the use of TXA, there remains a gap in the literature regarding its utility in panniculectomy and abdominoplasty. In the first review on the use of TXA in panniculectomy, the authors found no statistically significant reduction in hematoma, seroma, or drain duration when TXA was used in panniculectomy surgery. Collaborative work through large randomized controlled studies will help to define the role of this pharmacologic adjunct in body contouring surgery.
